# Supplementary material for: Epitope mapping of vaccine antigens Tc24 and TSA1 with antibodies from Trypanosoma cruzi-infected patients
Source: Genes Immun. 2026 Feb 10;27(2):195–202. doi: 10.1038/s41435-026-00380-8 (PMC13086574; doi:10.1038/s41435-026-00380-8)
Supplement: Supplementary file 1 — Supplementary Table 1 [file 41435_2026_380_MOESM1_ESM.docx]

**Supplementary Table 1. *T. cruzi* genomes used**

| **Strain** | **DTU** | **Country** | **Host** | **Source** |
| --- | --- | --- | --- | --- |
| Arequipa | TcI | Peru | Human | GCA_003594685.1 |
| Brazil_A4 | TcI | Brazil | Rat | GCA_015033625.1 |
| CGl14 | TcI | Colombia | Human | SRX1851527 |
| Corpus_Christi | TcI | USA | Human | SRX1054555 |
| Dm28c | TcI | Colombia | Opossum | GCA_003177105.1 |
| G_strain | TcI | Brazil | Opossum | GCA_003719455.1 |
| H1Yuc | TcI | Mexco | Human | SRX1851500 |
| Silvio_X10 | TcI | Brazil | Human | GCA_000188675.2 |
| TBM3324 | TcI | Ecuador | Triatomine | SRR3676267 |
| TcLM58 | TcI | USA | Macaque | Unpublished |
| TD25 | TcI | USA | Triatomine | SRR3676273 |
| WB1 | TcI | USA | Triatomine | Unpublished |
| Berenice | TcII | Brazil | Human | GCA_013358655.1 |
| Esmeraldo | TcII | Brazil | Human | GCA_000327425.1 |
| Ycl2 | TcII | Brazil | Human | GCA_003594485.1 |
| Ycl6 | TcII | Brazil | Human | GCA_015033655.1 |
| Ikiakarora | TcIII | Colombia | Triatomine | GCA_010117215.1 |
| Tc231 | TcIII | Brazil | Human | GCA_900252365.1 |
| M6241 | TcIII | Brazil | Human | SRR4023054, SRR4023055 |
| CanIII | TcIV | Brazil | Human | SRR1996498, SRR1996501 |
| Sum4Cl2 | TcIV | Mexico |  | Unpublished |
| Sum4Cl3 | TcIV | Mexico |  | Unpublished |
| TcGI52 | TcIV | USA | Macaque | Unpublished |
| TcMD12 | TcIV | USA | Macaque | Unpublished |
| 9280cl2 | TcV | Bolivia | Human | SRR1996492, SRR1996493, SRR1996496, SRR1996497, SRR1996502 |
| HE | TcV | Argentina | Human | Unpublished |
| SC43 | TcV | Bolivia | triatoma | GCA_015455285.1 |
| CL | TcVI | Brazil | Human | GCA_003719155.1 |
| CL_Brener | TcVI | Brazil | Human | GCA_000209065.1 |
| H1_Panama | TcVI | Panama | Human | Unpublished |
| TCC | TcVI | Chile | Human | GCA_003177095.1 |
| VD | TcVI | Argentina | Human | Unpublished |
