# Supplementary material for: Epitope mapping of vaccine antigens Tc24 and TSA1 with antibodies from Trypanosoma cruzi-infected patients
Source: Genes Immun. 2026 Feb 10;27(2):195–202. doi: 10.1038/s41435-026-00380-8 (PMC13086574; doi:10.1038/s41435-026-00380-8)
Supplement: Supplementary file 2 — Supplementary Table 2 [file 41435_2026_380_MOESM2_ESM.docx]

**Supplementary Table 2. Patient epitope recognition profile and *T. cruzi* diagnostic.**

|  | Dominant epitope recognition profile (N=20) | Alternative epitope recognition profile (N=7) | Significance |
| --- | --- | --- | --- |
| Age (years) | 25.3 ± 1.4 | 23.1 ± 2.4 | t=0.78, P=0.44 |
| Parity | 1.8 ± 0.9 | 0.7 ± 0.8 | t=1.2, P=0.24 |
| Stat-Pak + | 14 (0.70) | 5 (0.71) |  |
| Stat-Pak - | 6 (0.30) | 2 (0.29) | Χ^2^=0.005; d.f.=1; P=0.94 |
| T-detect + | 11 (0.55) | 5 (0.71) |  |
| T-detect - | 9 (0.45) | 2 (0.29) | Χ^2^=0.59; d.f.=1; P=0.44 |
| ELISA + | 6 (0.30) | 3 (0.43) |  |
| ELISA - | 14 (0.70) | 4 (0.57) | Χ^2^=0.38; d.f.=1; P=0.54 |
| ELISA OD* | 0.723 ± 0.246 | 0.811 ± 0.417 | t=0.2, P=0.86 |
| Confirmed seropositive^$^ | 7 (0.35) | 3 (0.43) |  |
| Serodiscordant | 13 (0.65) | 4 (0.57) | Χ^2^=0.14; d.f.=1; P=0.71 |
| PCR + | 19 (0.95) | 6 (0.86) |  |
| PCR - | 1 (0.05) | 1 (0.14) | Χ^2^=0.58; d.f.=1; P=0.45 |
| Parasite burden^#^ | 4.08 ± 0.72 | 2.54 ± 0.73 | t=1.5, P=0.24 |

*Optical density reading at 450 nm (mean ± SEM). ^$^Confirmed seropositive was defined as with 2-3 reactive serological tests, serodiscordant as with 0-1 reactive serological test but *T. cruzi* PCR positive. ^#^parasite burden in expressed as parasite equivalent/ml of blood (mean ± SEM).
