# Supplementary material for: Epitope mapping of vaccine antigens Tc24 and TSA1 with antibodies from Trypanosoma cruzi-infected patients
Source: Genes Immun. 2026 Feb 10;27(2):195–202. doi: 10.1038/s41435-026-00380-8 (PMC13086574; doi:10.1038/s41435-026-00380-8)
Supplement: Supplementary file 3 — Supplementary Table 3 [file 41435_2026_380_MOESM3_ESM.docx]

**Supplementary Table 3. HLA allele supertype frequency in patients according to epitope recognition profile of antigens.**

| **HLA frequencies** |  | **Dominant epitope profile (N=17)** | **Alternative epitope profile (N=6)** |
| --- | --- | --- | --- |
| HLA-A | A1 | 0.028 | 0.083 |
|  | A2 | 0.194 | 0.167 |
|  | A3 | 0.028 | 0 |
|  | A11 | 0.056 | 0 |
|  | A23 | 0 | 0.083 |
|  | A24 | 0.111 | 0.250 |
|  | A30 | 0.028 | 0 |
|  | A31 | 0.194 | 0.083 |
|  | A68 | 0.333 | 0.333 |
|  | A69 | 0.028 | 0 |
| HLA-B | B15 | 0.083 | 0.167 |
|  | B35 | 0.194 | 0.333 |
|  | B37 | 0 | 0.083 |
|  | B39 | 0.361 | 0.167 |
|  | B40 | 0.139 | 0.083 |
|  | B44 | 0 | 0.083 |
|  | B45 | 0.028 | 0 |
|  | B47 | 0.028 | 0 |
|  | B48 | 0.056 | 0 |
|  | B49 | 0.028 | 0 |
|  | B50 | 0.028 | 0 |
|  | B51 | 0.028 | 0 |
|  | B53 | 0.028 | 0 |
|  | B58 | 0 | 0.083 |
| HLA-C | C1 | 0.056 | 0.167 |
|  | C2 | 0 | 0.083 |
|  | C3 | 0.194 | 0.333 |
|  | C4 | 0.139 | 0.083 |
|  | C6 | 0.056 | 0.083 |
|  | C7 | 0.417 | 0.167 |
|  | C8 | 0.056 | 0 |
|  | C16 | 0.083 | 0.083 |
| HLA-DPA1 | DPA1 | 0.889 | 0.917 |
|  | DPA2 | 0.111 | 0.083 |
| HLA-DQA | DQA1 | 0.111 | 0 |
|  | DQA2 | 0.028 | 0.083 |
|  | DQA3 | 0.556 | 0.500 |
|  | DQA4 | 0.139 | 0 |
|  | DQA5 | 0.167 | 0.417 |
| HLA-DQB | DQB2 | 0.056 | 0.250 |
|  | DQB3 | 0.667 | 0.750 |
|  | DQB4 | 0.167 | 0 |
|  | DQB5 | 0.083 | 0 |
|  | DQB6 | 0.028 | 0 |
| HLA-DRB | DRB1 | 0.056 | 0 |
|  | DRB4 | 0.556 | 0.500 |
|  | DRB7 | 0.028 | 0.083 |
|  | DRB8 | 0.139 | 0 |
|  | DRB11 | 0.056 | 0 |
|  | DRB13 | 0.056 | 0.083 |
|  | DRB14 | 0.111 | 0.167 |
|  | DRB16 | 0 | 0.167 |
| HLA-DRB345 | DRB345-1 | 0.862 | 0.667 |
|  | DRB345-2 | 0.103 | 0.250 |
|  | DRB345-10 | 0 | 0.083 |
|  | DRB345-13 | 0.034 | 0 |
