# Supplementary material for: Epitope mapping of vaccine antigens Tc24 and TSA1 with antibodies from Trypanosoma cruzi-infected patients
Source: Genes Immun. 2026 Feb 10;27(2):195–202. doi: 10.1038/s41435-026-00380-8 (PMC13086574; doi:10.1038/s41435-026-00380-8)
Supplement: Supplementary file 4 — Supplementary Figure 1 [file 41435_2026_380_MOESM4_ESM.pdf]

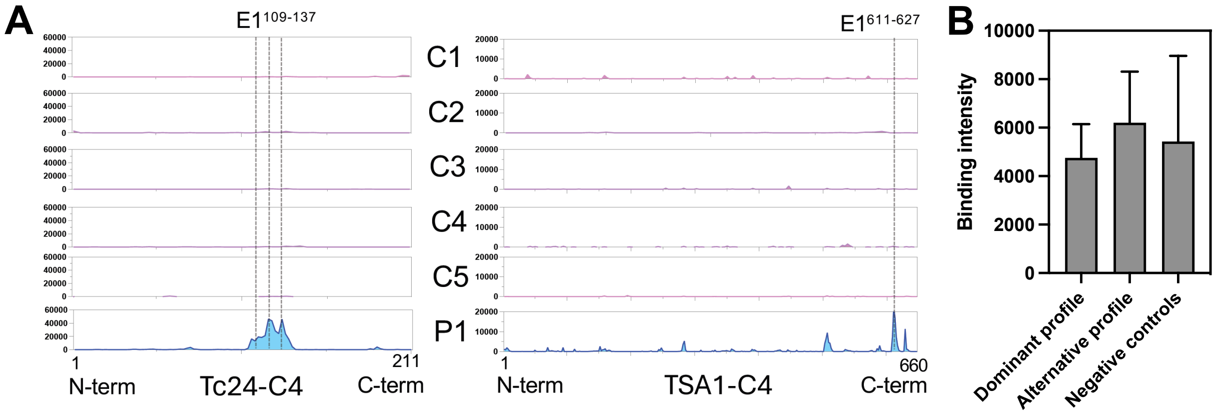

Supplementary Figure 1. Microarray binding specificity and assay validation. (**A**) Overlapping peptides covering the full length of the primary sequence for Tc24-C4 (left) and TSA1-C4 (right), horizontal axis, were evaluated in microarrays with IgGs from five uninfected control participants (C1 to C5). One *T. cruzi* infected patient (P1) is shown for comparison. The main epitope (E1) is indicated for both proteins. (**B**) Recognition of the Herpes control peptide among subgroups of participants. *T. cruzi* infected patients are divided into dominant and alternative antibody profiles (see main text for explanations). There was no significant difference in control peptide binding among these groups (ANOVA, F=0.11, P=0.89).
